# CTD small phosphatase like 2 (CTDSPL2) can increase ε- and γ-globin gene expression in K562 cells and CD34+ cells derived from umbilical cord blood

**DOI:** 10.1186/1471-2121-11-75

**Published:** 2010-10-09

**Authors:** Yan-Ni Ma, Xin Zhang, Hai-Chuan Yu, Jun-Wu Zhang

**Affiliations:** 1 National Laboratory of Medical Molecular Biology, Institute of Basic Medical Sciences, Chinese Academy of Medical Sciences and Peking Union Medical College, 5 Dong Dan San Tiao, Beijing 100005, People's Republic of China

## Abstract

**Background:**

A potential strategy for treatment of sickle cell disease (SCD) and β-thalassemia in adults is reactivation of the ε- and γ-globin genes in the adult. We aimed to identify trans-activators of ε- and γ-globin expression and provide new candidate targets for effective treatment of sickle cell disease (SCD) and β-thalassemia through activation of ε- and γ-globin genes in adults.

**Results:**

We identified a CTD small phosphatase like 2 (CTDSPL2) gene that had higher transcription levels in umbilical cord blood (UCB) than in adult bone marrow (BM). Also, transcription of the CTDSPL2 gene increased significantly during erythroid differentiation. Further, we found that overexpression of CTDSPL2 could obviously improve the expression of ε- and γ-globin genes in K562 cells. Meanwhile, the repression of CTDSPL2 by RNA interference decreased expression of ε- and γ-globin genes but did not inhibit the increase of globin gene expression during K562 erythroid differentiation. In addition, the enforced expression of CTDSPL2 gene mediated by lentiviruses could also increase ε- and γ-globin gene expression during erythroid differentiation of CD34+ cells derived from UCB.

**Conclusion:**

CTDSPL2 gene can obviously improve the expression of ε- and γ-globin genes in K562 cells and CD34+ cells derived from UCB. Our study provides a new candidate target for effective treatment of SCD and β-thalassemia.

## Background

During development the expression of human β-like globin genes displays two switches: the embryonic (ε-) to fetal (Gγ- and Aγ-) globin switch, coinciding with the transition from embryonic (yolk sac) to definitive (fetal liver) haematopoiesis, and the fetal to adult (β-) globin switch, occurring near the parturient period with the establishment of bone marrow as the main site of hematopoiesis [1,2]. It has been shown that fetal hemoglobin (HbF, α_2_γ_2_) expression can be reactivated during adult erythropoiesis [3]. The increased production of fetal hemoglobin could ameliorate the clinical severity of sickle cell anemia (SCD) and β-thalassemia [3]. Therefore, attempts are underway to screen for pharmaceuticals that reactivate γ-globin production in adults. Hydroxyurea and sodium butyrate derivatives have been already been examined and used for treatment of β-hemoglobinopathies [4-8]. While these therapies are effective for treating SCD and β-thalassemia, they also have adverse effects such as suppression of cell growth and bad effects in long-term treatment [9,10].

An alternative treatment strategy is to increase γ-globin expression by controlling potential transcription factors that specifically activate γ-globin expression in the fetus or result in γ-globin gene silencing in the adult. To identify genes encoding such factors, we analyzed differential expression of mRNA in erythroid induction cultures of CD34+ hematopoietic progenitor cells derived from normal adult bone marrow (BM) and umbilical cord blood (UCB). One of the differentially expressed genes was further examined for effects on globin gene expression.

Expression levels of the CTD small phosphatase like 2 (CTDSPL2) gene were higher in UCB than in healthy adult BM. CTD phosphatase dephosphorylates the CTD tail of the largest subnit of RNA polymerase II (RNAPII). *In vivo*, the C-terminal domain (CTD) of the largest subunit of RNA polymerase II is either phosphorylated or dephosphorylated. Dynamic, site-specific phosphorylation/dephosphorylation of the CTD occurs during the transcription cycle [11]. Recent observations indicate that CTD phosphorylation plays a major role in orchestrating interaction of the CTD with mRNA processing factors involved in capping, splicing and polyadenylation. Several kinases modify the CTD phosphorylation state. However, only a few mammalian CTD phosphatases have been identified. FCP1, the first characterized CTD phosphatase, contains a BRCT domain required for RNAPII interaction and CTD dephosphorylation [12]. SCP1 and UBLCP1 contain phosphatase domains similar to FCP1, and also dephosphorylate Ser-2 and Ser-5 of phosphorylated CTD *in vitro *[13]. The CTDSPL2 gene product contains a CTD phosphatase catalytic domain (CPDc) and the gene is 47% homologous to FCP1. The function of this gene is not known although it has been shown to dephosphorylate phophoserine-5 and phosphoserine-2 within GST-CTDo *in vitro *[14].

In this study, we show that overexpression of CTDSPL2 significantly increased the ε- and γ- globin gene expression in K562 cells and CD34+ cells derived from UCB while have faint effect on ζ-, α- and β-globin gene.

## Results

### Expression pattern of CTDSPL2 in BM and UCB and during erythroid differentiation

**Figure 1:**
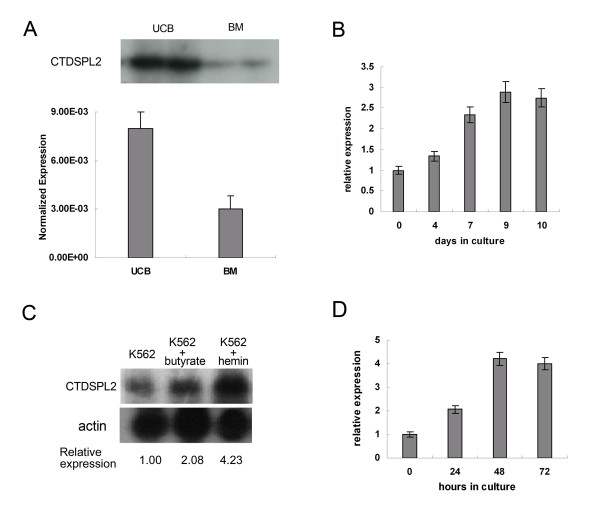
**CTDSPL2 mRNA levels in erythroid cultures from BM and UCB and during erythroid differentiation of K562 cells and CD34+ HPCs**. **(A) **Differential expression of CTDSPL2 in erythroid cultures from healthy adult bone marrow (BM) and umbilical cord blood (UCB) as measured by DDRT-PCR (top) and real-time PCR (bottom). **(B) **CTDSPL2 mRNA expression at different days during EPO-induced CD34+ cell erythroid differentiation as determined by real-time PCR. **(C) **Northern blot analysis of CTDSPL2 mRNA expression in K562 cells 48 h after treatment with butyrate and hemin. **(D) **Real-time PCR analysis of CTDSPL2 mRNA expression at different time points during hemin-induced K562 cell erythroid differentiation.

We analyzed differential expression of genes in CD34+ erythroid induction cell cultures derived from normal adult BM and UCB by differential display reverse transcription PCR (DDRT-PCR). CTDSPL2 had higher expression levels in erythroid cultures derived from UCB than from healthy adult BM. Differential expression of CTDSPL2 was also detected by real-time PCR. Expression levels of CTDSPL2 mRNA in UCB erythroid cultures were 2.6 times greater than in healthy adult BM erythroid cultures (Fig. 1A).

The level of CTDSPL2 mRNA during Epo-induced erythroid differentiation in UCB-derived CD34+ HPCs was measured by real-time PCR. CTDSPL2 mRNA levels increased during erythroid differentiation (Fig. 1B). The mRNA expression of CTDSPL2 in hemin- and butyrate-induced K562 cells was measured by Northern blot analysis (Fig. 1C). CTDSPL2 mRNA levels were also measured by real-time PCR at different time points during hemin-induction of K562 cells (Fig. 1D). Levels of CTDSPL2 mRNA increased in hemin- and butyrate-induced K562 cells. CTDSPL2's increase was more evident during hemin-induced K562 cell differentiation.

### Overexpression of CTDSPL2 increases hemoglobin-containing cells during erythroid differentiation of K562 cells

**Figure 2:**
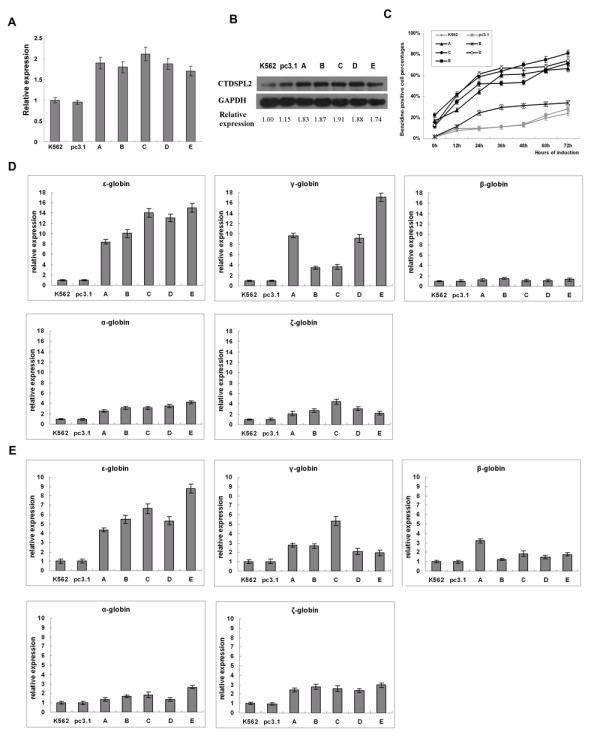
**Overexpression of *CTDSPL2 *increases ε- and γ- globin gene expression in K562 cells**. **(A) **Overexpression of CTDSPL2 mRNA in stable K562/pc3.1-CTDSPL2 transfectant pools as analyzed by real-time PCR. **(B) **Overexpression of CTDSPL2 protein in stable K562/pc3.1-CTDSPL2 transfectant pools as analyzed by Western blot. **(C) **Hemoglobin-containing cells were detected by benzidine staining during hemin-induced erythroid differentiation of K562 cells. The data were obtained from three independent experiments and the error bars represent standard deviation. **(D) **Real-time PCR analysis of α-, ζ-, ε-, γ- and β-globin genes mRNA expression in K562 cells, K562/pc3.1 transfectants and K562/pc3.1-CTDSPL2 transfectants. **(E) **Real-time PCR analysis of α-, ζ-, ε-, γ- and β-globin mRNA expression in K562 cells, stable K562/pc3.1 transfectants and K562/pc3.1-CTDSPL2 transfectants 72 h after treatment with hemin. K562 and pc3.1 represent K562 cells and stable K562/pc3.1 transfectant pool respectively. A, B, C, D and E represent the five stable K562/pc3.1-CTDSPL2 transfectant pools respectively.

We collected five stable pools of K562 cells transfected with pc3.1-CTDSPL2 (each pool was originally from a collection of 20 to 30 individual clones). Real-time PCR analysis revealed that transcription of the CTDSPL2 gene in these five transfectant pools was on average 2 times greater than in the K562/pc3.1 transfectant pools and in un-transfected K562 cells (Fig. 2A). The Western blot analysis also revealed an obvious increase of CTDSPL2 protein in the K562/pc3.1-CTDSPL2 transfectant pools (Fig. 2B).

Erythroid differentiation was induced in stable K562 transfectants and un-transfected K562 cells by hemin. The cells were collected and analyzed by benzidine staining 0, 12, 24, 36, 48, 60, 72 h after hemin induction (Fig. 2C). Among the five pc3.1-CTDSPL2 transfectant pools, there were four (A, C, D and E) with especially high benzidine-positive cell levels. Before erythroid differentiation, 17-22% of the cells were benzidine-positive, which is equivalent to that in control cells 60 h after being induced by hemin. For these four transfectant pools, benzidine-positive cells were 75-80% at 72 h after hemin induction, significantly greater than for control cells. The percentage of benzidine-positive cells in the transfectant pool B was also higher than control cells during erythroid differentiation.

### Overexpression of CTDSPL2 increases ε- and γ- globin gene expression in K562 cells

The transcription of a number of globin genes (including α-, ζ-, ε-, γ- and β-globin genes) was analyzed by real-time PCR in the K562 transfectants. Overexpression of the CTDSPL2 gene significantly increased transcription of ε- and γ-globin genes in K562 cells but had little effect on α-, ζ- and β-globin gene transcription. Transcription of ε- and γ-globin genes in the stable K562 pc3.1-CTDSPL2 transfectant pools was 12 and 6 times greater respectively than K562/pc3.1 transfectant pools and untransfected K562 cells (Fig. 2D). The improvement on α-, ζ- and β-globin gene expression was weak relative to the effects on ε- and γ-globin genes.

Erythroid differentiation in the transfected cells was induced by hemin. Cells were collected 72 h after hemin induction. Overexpression of CTDSPL2 increased expression of ε- and γ-globin genes after erythroid differentiation of K562 cells (Fig. 2E). The expression of ε- and γ-globin gene was 7 and 3.5 times greater respectively in the K562 transfectants overexpressing CTDSPL2 than in K562/pc3.1 control cells and K562 cells after erythroid differentiation. The effects of CTDSPL2 overexpression on globin gene transcription were less before erythroid differentiation than after differentiation.

**Figure 3:**
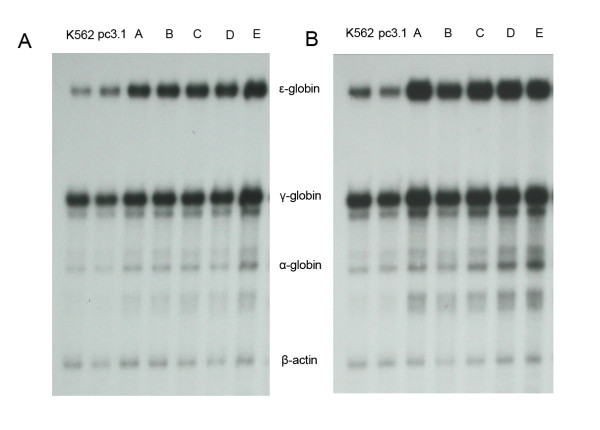
**RNase protective assay of α-, ε-, γ- and β-globin gene mRNA expression in K562 cells before and after erythroid differentiation**. **(A) **Globin gene expression in un-induced K562 cells. **(B) **Globin gene expression in K562 cells 72 h after treatment with hemin. K562 and pc3.1 represent K562 cells and stable K562/pc3.1 transfectant pool respectively. A, B, C, D and E represent the five stable K562/pc3.1-CTDSPL2 transfectant pools respectively.

Transcription of α-, ε-, γ- and β-globin genes in the transfected K562 cells before and after erythroid differentiation was also analyzed by RNase protective assay. Before hemin induction (Fig. 3A), ε-globin mRNA levels in the stable K562/pc3.1-CTDSPL2 transfectants were significantly higher than in control cells. γ-globin mRNA levels in K562/pc3.1-CTDSPL2 transfectants were also greater relative to control cells which have high γ-globin mRNA levels. α-globin mRNA in K562/pc3.1-CTDSPL2 transfectants was greater than in control cells but α-globin mRNA was still at a low level. β-globin mRNA was undetected in all samples.

After hemin induction, transcription of all globin genes excluding β-globin increased in all cells (Fig. 3B). Levels of ε- and γ-globin mRNA in the stable K562/pc3.1-CTDSPL2 transfectants was higher than in control cells. Transcription of the α-globin gene in the stable K562/pc3.1-CTDSPL2 transfectants was also higher than in the control cells but the α-globin mRNA was still at a low level. β-globin mRNA was still undetected after erythroid differentiation.

### Repression of CTDSPL2 gene decreases the number of hemoglobin-containing K562 cells

**Figure 4:**
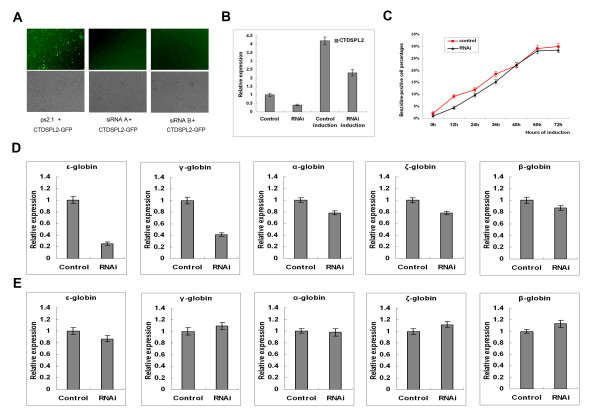
**Effects of CTDSPL2 repression on globin gene expression in K562 cells**. **(A) **Fluorescent microscopy indicating CTDSPL2-GFP expression showing CTDSPL2 repression by two siRNAs in Hela cells. **(B) **Real-time PCR analysis of CTDSPL2 mRNA expression in stable K562/CTDSPL2 RNAi transfectants. **(C) **Hemoglobin-containing cells were detected by benzidine staining during hemin-induced erythroid differentiation of the above K562 cells. The data were obtained from three independent experiments and the error bars represent standard deviation. **(D) **Real-time PCR analysis of α-, ζ-, ε-, γ- and β-globin mRNA expression in the stable K562/CTDSPL2RNAi transfectants carrying the recombinant pLVTHM CTDSPL2RNAi (RNAi) and the stable K562/CTDSPL2 transfectants carrying the blank pLVTHM (Control). **(E) **Real-time PCR analysis of α-, ζ-, ε-, γ- and β-globin mRNA expression 72 h after hemin treatment in stable K562/CTDSPL2RNAi transfectants and the stable K562/CTDSPL2 transfectants.

Recombinant RNAi plasmids (RNAiA, RNAiB) and the pSilencer2.1-U6-Neo control vector were co-transfected into Hela cells with the CTDSPL2-GFP fusion expression plasmid. CTDSPL2-GFP expression was observed to confirm CTDSPL2 repression in the cells. Expression of CTDSPL2-GFP was strongly repressed in Hela cells transfected with RNAiA and RNAiB compared to cells transfected with pSilencer2.1-U6-Neo (Fig. 4A). Both the intensity of green fluorescence in each cell and the number of cells that express green fluorescent protein decreased. RNAiB transfection resulted in greater repression of fluorescence. These results indicate that these two RNAi plasmids repress expression of CTDSPL2-GFP effectively. As the repression efficiency of RNAiB was much greater, RNAiB (1057 AAGGTGTATGCAGACAAGTTA) was used in further experiments.

RNAiB was subcloned into the pLVTHM vector and high titer lentiviruses were acquired. By lentivirus infection, we got stable K562/CTDSPL2RNAi transfectant pool. CTDSPL2 mRNA level measured by real-time PCR in the stable K562/CTDSPL2 RNAi transfectant pool was 40% of that in the control cells (Fig. 4B). An increased CTDSPL2 mRNA level in the stable K562/CTDSPL2 RNAi transfectant pool was detected after hemin induction 72 h but it was only 55% of that in the hemin-induced control cells.

Erythroid differentiation was induced in stable K562/CTDSPL2 RNAi transfectant pool and control cells by hemin and the cells were collected and analyzed by benzidine staining at different time points after hemin induction (Fig. 4C). Benzidine-positive cell percentages in stable K562/CTDSPL2 RNAi transfectant pool were lower than in control cells at the early erythroid differentiation stage. However, this phenomenon disappeared after hemin induction 48 h. The phenomena suggested that the repression of CTDSPL2 gene in K562 cells could decrease the hemoglobin-containing cells but could not inhibit the increase of hemoglobin-containing cells during hemin-induced K562 cells erythroid differentiation.

### Repression of CTDSPL2 gene in K562 cells can decrease the expression of ε- and γ-globin gene

The transcription of α-, ζ-, ε-, γ- and β-globin genes in the stable K562/CTDSPL2 RNAi transfectants and control cells were analyzed by real-time PCR before and after erythroid differentiation. Before hemin induction, ε- and γ-globin transcription in the K562/CTDSPL2 RNAi transfectants was much less than that in control cells (Fig. 4D). ε-globin gene transcription was only 25% of that in control cells while γ- globin transcription was 41% of that in control cells. β-, ζ- and β-globin gene transcription was also less than that in control cells but the difference was not significant. 72 h after hemin induction, no significant difference of any globin gene transcription was found between the stable K562/CTDSPL2 RNAi transfectants and control cells (Fig. 4E).

### CTDSPL2 overexpression increases ε- and γ- globin gene expression in CD34+ HPCs derived from UCB

**Figure 5:**
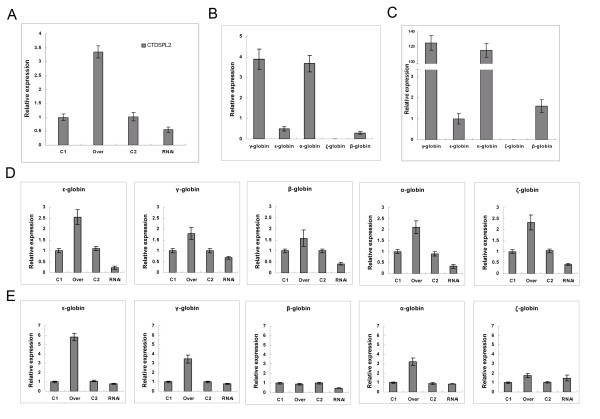
**Effects of overexpression and repression of CTDSPL2 on globin gene expression in CD34+ HPCs**. **(A) **Detection of overexpression and repression of *CTDSPL2 *gene in CD34+ cells by real-time PCR. **(B) **Relative expression of globin genes in CD34+ cells derived from UCB. **(C) **Relative expression of globin genes in CD34+ cells derived from UCB 7 days after Epo-induced erythroid differentiation. **(D) **Real-time PCR analysis of globin gene expression in CD34+ cells overexpressing or repressing CTDSPL2. **(E) **Real-time PCR analysis of globin genes in EPO-induced CD34+ cells overexpressing or repressing CTDSPL2. C1, Over, C2, RNAi represent the CD34+ cells infected respectively with lentiviruses carrying the blank pWPXL vector, recombinant pWPXL-CTDSPL2, blank pLVTHM vector and recombinant pLVTHM-CTDSPL2RNAi.

Two high titer lentiviruses that carry pWPXL-CTDSPL2 and pLVTHM-CTDSPL2 RNAi were obtained. CD34+ HPCs from normal UCB were purified and were infected with the two recombinant lentiviruses respectively. The transduction efficiency of the CD34+ cells was about 70-80% through observing GFP expression. CTDSPL2 mRNA was detected by real-time PCR. CTDSPL2 mRNA levels in CD34+ cells infected by lentiviruses carrying pWPXL-CTDSPL2 were 3.5 times greater than that in control cells (Fig. 5A). CTDSPL2 mRNA levels in CD34+ cells infected by lentiviruses carrying pLVTHM-CTDSPL2 RNAi were 55% of that in control cells (Fig. 5A).

Globin mRNA levels in the lentivirus-infected CD34+ cells were analyzed by real-time PCR before and after Epo-induced erythroid differentiation. There were mainly the α- and γ-globin gene expression in UCB derived CD34+ cells (Fig. 5B). α- and γ-globin mRNA levels were 40 times greater after Epo-induced erythroid differentiation (Fig. 5C).

Overexpression of CTDSPL2 gene in CD34+ HPCs increased transcription of all globin genes (Fig. 5D). ε-globin mRNA in CD34+ HPCs overexpressing CTDSPL2 was 2.5 times greater than in control cells. Repression of the CTDSPL2 gene in CD34+ HPCs decreased transcription of the ε-globin gene but had little effect on other globin genes. ε-globin gene expression in CD34+ HPCs with repressed CTDSPL2 was only 22% of that in control cells.

Transcription of a number of globin genes in the lentivirus-infected CD34+ cells was measured 7 days after induction by Epo (Fig. 5E). Increases in ε-globin gene transcription in Epo-induced CD34+ cells were much stronger than in un-induced CD34+ cells. ε-globin gene transcription in CD34+ HPCs overexpressing CTDSPL2 was 5.8 times greater than in control cells. Overexpression of the CTDSPL2 gene also increased α- and γ-globin gene transcription in Epo-induced CD34+ cells. Decreases of globin gene transcription in CD34+ cells with repressed CTDSPL2 were no longer observed after erythroid differentiation.

## Discussion

By differential display analysis, we found that the transcription of the CTDSPL2 gene in UCB erythroid cultures was greater than in adult BM erythroid cultures. Meanwhile, γ-globin gene developmentally expressed mainly in UCB and little in adult BM [15]. Furthermore, we measured transcription levels of CTDSPL2 during hemin-induced erythroid differentiation of K562 cells and Epo-induced erythroid differentiation of CD34+ HPCs and found that its expression increased significantly during erythroid differentiation. γ-globin gene transcription also increased significantly during hemin-induced erythroid differentiation in K562 cells and Epo-induced erythroid differentiation in CD34+ HPCs [16,17]. Based on this evidence, we hypothesized that CTDSPL2 plays a role in γ-globin gene activation.

CTD phosphatases can dephosphorylate the CTD tail of the largest subnit of RNAPII and play a major role in mRNA processing. Only a few mammalian CTD phosphatases have been identified [12]. CTD small phosphatase like 2 (CTDSPL2) contains a CTD phosphatase catalytic domain (CPDc) and dephosphorylates phophoserine-5 and phosphoserine-2 within GST-CTDo *in vitro *[14].

The K562 cell line was chosen for this study as it is commonly used to study globin gene regulation [18,19]. We prepared five stable K562/pc3.1-CTDSPL2 transfectant pools. Benzidine staining showed that overexpression of the CTDSPL2 gene increased the number of hemoglobin-containing cells during K562 erythroid differentiation. To determine which globin genes are affected, a number of globin genes (including α-, ζ-, ε-, γ- and β-globin genes) were analyzed. The results showed that overexpression of CTDSPL2 increased the transcription of ε- and γ-globin genes in K562 cells before and after erythroid differentiation, while there was only a small effect on the α-, ζ- and β-globin genes. Of course, the random integration into genome and the incomplete simulation of endogenous gene in overexpression study may arise some trustless results. But the use of several stable transfectant pools in this study may partially overcome this disadvantage.

CTDSPL2 may be specifically recruited into the transcriptional initiation complex at the ε- and γ-globin gene promoters through interaction with ε- and γ-globin specific transcription factors. In order to identify candidate transcription factors, CTDSPL2-interacting proteins will need to be determined. Expression of the erythroid surface marker, CD71, increased to some degree in the stable K562/pc3.1-CTDSPL2 transfectant pools (data not shown). So, it is also possible that CTDSPL2 increased ε- and γ-globin gene expression by increasing erythroid differentiation of K562 cells partially.

To determine if CTDSPL2 is necessary for ε- and γ-globin gene expression, we examined the effect of CTDSPL2 repression by RNAi on globin gene expression in K562 cells. Repression of the CTDSPL2 gene decreased the transcription of the globin genes, especially the ε-globin gene. However, repression of CTDSPL2 did not inhibit the increase of globin gene expression during hemin-induced erythroid differentiation. Therefore, CTDSPL2 can increase ε- and γ-globin gene expression but it is not indispensable for increases of ε- and γ-globin gene expression during erythroid differentiation. There may be other genes that can compensate for CTDSPL2 in the activation of globin gene expression. Alternatively, CTDSPL2 level is decreased but not eliminated in our experiments. Transcription of CTDSPL2 in stable K562/CTDSPL2 RNAi transfectants still increased during erythroid differentiation. This may explain why repression of CTDSPL2 could not inhibit the increase of globin gene expression during erythroid differentiation.

The effects of CTDSPL2 on globin gene expression in CD34+ hematopoietic progenitor cells were also examined. CD34+ hematopoietic progenitor cells were quiescent and generally non-dividing. Lentiviruses can effectively infect quiescent, non-divisive cells and are widely used in hematopoietic stem cell gene therapy [20,21]. We achieved overexpression and repression of the CTDSPL2 gene in CD34+ cells by lentivirus infection. The effects in lentivirus-infected CD34+ cells were consistent with those in transfected K562 cells. CTDSPL2 overexpression significantly increased ε-globin gene expression and also increased γ-globin gene transcription. The greater effect of CTDSPL2 on ε-globin gene transcription than γ-globin gene transcription may be partly due to the high baseline level of γ-globin gene transcription in K562 cells and UCB-derived CD34+ HPCs. In future studies, the effect of CTDSPL2 on γ-globin gene transcription will be measured in bone marrow-derived CD34+ HPCs in which there is little γ-globin gene expression.

The expression of CTDSPL2 increased significantly during erythroid differentiation. This suggested that its expression may be regulated by erythroid differentiation-related factors. The analysis of its promoter and mechanism of its specific expression is necessary. The overexpression of CTDSPL2 can specifically increase epsilon and gamma globin spontaneous expression, but CTDSPL2 does not contain a DNA binding domain. So it is impossible for CTDSPL2 to bind to epsilon and gamma-globin gene promoter directly. And it is possible that it was recruited to epsilon and gamma-globin gene promoter by interacting with some important globin related transcriptional factors such as EKLF, FKLF, NF-E4, etc. So to find the possible CTDSPL2 interacting protein through immunoprecipitation or two hybrid system in mammalian cell is the next step. In addition to identify the existence of CTDSPL2 in epsilon and gamma globin gene promoter complex through chromatin immunoprecipitation is also necessary. On the other hand, it is possible that CTDSPL2 regulate the phosphorylation status of the key factors in globin gene expression. It is reported that SCP1 can dephosphorylate Ser-2 and Ser-5 of phosphorylated RNA polII CTD *in vitro* as FCP1 (the first characterized CTD phosphatase) [13]. It is also reported that SCP1-3 can dephosphorylate Smad1 C-terminal tail, thereby attenuating BMP signaling [22]. It also can dephosphorylate the linker regions of Smad1 and Smad2/3 *in vitro*[23]. So the substrate of CTDSPL2 may be various not limited to the CTD tail of RNA polII.

## Conclusions

In conclusion this study showed that CTDSPL2 increases ε- and γ-globin gene expression in K562 cells and UCB-derived CD34+ HPCs. Reactivation of the γ-globin gene in the adult has been demonstrated to be significant for treatment of SCD and β-thalassemia. The reactivation of the ε-globin gene should also have similar effects. Thus our study provides a new candidate target for effective treatment of SCD and β-thalassemia through activation of ε- and γ-globin genes in adults. Further investigation of the mechanism of CTD phosphatase in globin gene regulation may open new ways to regulate gene transcription with CTD phosphatases.

## Methods

### Cell culture and induction to erythroid differentiation

Informed consent was obtained from all donors. Four UCB samples (40 to 50 ml each) from normal full-term deliveries and four BM samples (5 to 10 ml each) from healthy adult donors were collected using EDTA as the anti-coagulant. UCB samples were diluted 1:4 with phosphate buffered saline (PBS) containing 2 mM EDTA and 0.5% bovine serum albumin (BSA, Sigma, St. Louis, MO, USA). To release the cells, BM samples were diluted with 10 volumes of RPMI 1640 (Gibco, Invitrogen, Grand Island, NY, USA) containing 0.02% collagenase B and 100 U/ml DNase (Sigma, St. Louis, MO, USA) and incubated at room temperature for 45 min. Mononuclear cells (MNCs) were isolated from the diluted UCB and BM by centrifugation on a gradient of Ficoll-Hypaque (density 1.077 g/ml, Sigma, St. Louis, MO, USA) [24]. CD34+ HPCs were purified by positive selection using Mini-MACS columns according to the manufacturer's protocol (Milternyi Biotech, Germany). The purity of CD34+ cells was then determined (> 90%).

Cells were cultured as described previously [25]. Briefly, CD34+ HPCs purified from UCB were cultured in Iscove's modified Dulbecco's medium (IMDM, Gibco, Invitrogen, Grand Island, NY, USA) containing 1% deionized bovine serum albumin (BSA, fraction V, Sigma, St. Louis, MO, USA), 30% human cord serum (CHS, prepared in-house, heterologous), 1 × 10^-5 ^mol/L 2-mercaptoethanol (Sigma, St. Louis, MO, USA), 2 × 10^-3 ^mol/L L-glutamine (Sigma, St. Louis, MO, USA), antibiotics (Gibco, Invitrogen, Grand Island, NY, US) and cytokines (R&D System, Minneapolis, MN. USA) including 2 IU/ml Epo, 100 ng/ml SCF and 2 ng/ml IL-3. CD34+ cells from BM were cultured in the same serum-containing medium except that 30% CHS was substituted with 30% human adult AB-type serum (AHS, TBD, Tianjin, P R China). All CD34+ cells were seeded at 1 × 10^5 ^cells/ml in 6 ml cultures and incubated in a humidified atmosphere at 37°C and 5% CO_2 _for three weeks.

K562 cells were cultured in DMEM medium (Gibco, Invitrogen, Grand Island, NY, USA) containing 10% fetal bovine serum and 2 × 10^-3 ^mol/L L-glutamine at 37°C and 5% CO_2 _and were induced to erythroid differentiation with 40 μmol/L hemin [26]. Cells were counted with a hemocytometer and hemoglobin-containing erythroid cells were identified by benzidine staining.

### RNA preparation, cDNA synthesis and real-time PCR

Total RNA was extracted from cell samples using TRIzol^® ^reagent (Invitrogen, Carlsbad, CA, USA) and quantitated by spectrophotometer. The first-strand of cDNA was synthesized using Superscript™III reverse transcriptase (Invitrogen, Carlsbad, CA, USA). Gene-specific primers were designed using Primer Express (Applied Biosystems, Foster City, CA) software Version 2.0 (Table 1) [27]. The mRNA level of target genes in cultured erythroid cells was quantified by real-time PCR analysis on an ABI PRISM^® ^7500 real-time PCR System (Applied Biosystems, Foster City, CA) with the SYBR^® ^*Premix Ex Taq*™kit (Takara, Dalian, P R China). The following PCR cycle parameters were used: 95°C for 10 s, 40 cycles at 95°C for 5 s, 60°C for 34 s. Each PCR reaction was performed in triplex tubes with GAPDH as an endogenous control to standardize the amount of sample cDNA. Data were analyzed with sequence detection system (SDS) software (Applied Biosystems, Foster City, CA). The comparative C_T _method was used for quantification of the target genes relative to GAPDH. All real-time PCR analyses were repeated three times.

**Table 1:** Oligonucleotide primer sets used in quantitative real-time PCR experiments

*Gene*	*Primer Sequence (5'-3')*	*Product Size (bp)*
γ-globin	GCAGCTTGTCACAGTGCAGTTC TGGCAAGAAGGTGCTGACTTC	166
β-globin	GTCTACCCTTGGACCCAGAGGTTC TGAGCCAGGCCATCACTAAAG	131
ε-globin	CAGCTGCAATCACTAGCAAGC AGACGACAGGTTTCCAAAGC	190
α-globin	GGTCAACTTCAAGCTCCTAAGC GCTCACAGAAGCCAGGAACTTG	116
ζ-globin	TGAGCGAGCTGCACGCCTAC GTACTTCTCGGTCAGGACAGA	173
CTDSPL2	TGGAACGTCAGGATCAGATTCTC GATGGTCTCACTTGAACTGCTTGA	157
GAPDH	TCAACGACCACTTTGTCAAGCTCA GCTGGTGGTCCAGGGGTCTTACT	119

### Differential display reverse transcription PCR

CD34+ cells derived from UCB and adult BM were collected on day 8 of erythroid culture as our previous results showed that this is when γ- to β-globin switching occurs [25]. Total RNA was prepared as above and differential display reverse transcription-PCR (DDRT-PCR) was performed in combination with 3 anchored primers and 18 arbitrary primers. Briefly, three one-base anchored oligo-dT primers (A, C, G) were used to synthesize first-strand cDNA that was then amplified with 18 arbitrary primers (long primers) in the presence of [α-^32^P] dCTP. The following PCR cycle parameters were used: 94°C for 1 min, 40°C for 4 min, 72°C for 1 min; 35 cycles at 94°C for 1 min, 60°C for 2 min, 72°C for 1 min; 72°C for 5 min. Each PCR reaction was performed in double tubes to confirm the reproducibility and amplified with GAPDH as an endogenous control to standardize the amount of sample cDNA. PCR products were separated on a 6% denaturing polyacrylamide gel at 85 W on the gel board of sequencing (Bio-Rad California, USA) according to the manufacturer's instructions. The gel was analyzed using the Cyclone Storage Phosphor System (Packard, USA) and then exposed for 6 to 12 h at -70°C on X-film (Kodak, P.R China) after drying at 80°C for 120 min under vacuum (Bio-Rad California, USA).

### Western blot

Cell lysates containing 50 μg protein were loaded on a 12% SDS-PAGE gel and then were transferred to polyvinylidene difluoride membranes (Amersham) using Bio-Rad's Transblot for 2 h at 0.2 amp at 4°C. The membrane was immersed in the blocking buffer (PBS containing 3% BSA and 0.05% Tween 20) and blocked overnight. Then the membrane was incubated with the AntiCTDSPL2 (ProTeintech Group, Inc, Chicago, USA) or AntiGAPDH (Abcam, Cambridge, UK) diluted in blocking buffer for 2 h at room temperature, followed by incubated with the peroxidase-conjugated affinipure goat anit-mouse IgG (H+L) (Zhongshang Goldenbridge, Beijing, China) diluted in the blocking buffer for 2 h at room temperature. Then chemiluminescence reaction was performed using ECL Western Blotting Analysis System (Amersham) for 5 min, and then the membranes were exposed to ECL hyper film (Amersham) for 5 s-15 min.

### Overexpression of *CTDSPL2 *in K562 cells by lipofectamine transfection

The cDNA fragment including the complete CTDSPL2-coding sequence was subcloned into Hind III-EcoRV of the eukaryotic expression vector pcDNA3.1+ (Invitrogen, Carlsbad, CA, USA). K562 cells were transfected with the recombinant plasmid pc3.1-CTDSPL2 and pc3.1 vector as a control by Lipofectamine 2000 (Invitrogen, Carlsbad, CA, USA). Stable K562 transfectants were selected in the medium containing 500 μg/ml G418 for 2 weeks. Overexpression of CTDSPL2 in K562 cells was validated by real-time PCR.

### Screening for effective RNAi targets of *CTDSPL2 *gene

The CTDSPL2 open reading frame (ORF) was analyzed with the Ambion siRNA target finder http://www.ambion.com. Several possible targets for effective RNAi of CTDSPL2 gene were selected. DNA sequences that are siRNAs targets for two sites (326, AAGCTGGTAGTTATGAAATGA; 1057, AAGGTGTATGCAGACAAGTTA) were inserted into the psilencer2.1-U6-neo vector at the BamHI and HindIII sites. The coding sequence of CTDSPL2 gene excluding the stop codon was inserted into the eukaryotic expression vector pEGFP-N1 in frame of EGFP and to produce CTDSPL2-GFP fusion protein expression plasmid. Recombinant RNAi plasmids were then co-transfected with the CTDSPL2-GFP fusion expression plasmid into Hela cells. The green fluorescence was observed to validate CTDSPL2 repression in Hela cells.

### Recombinant lentiviruses generation and gene transduction in CD34+ HPCs

The coding sequence of the CTDSPL2 gene was subcloned into the pWPXL retroviral expression vector at BamHI and MluI sites joining the 5'end of EGFP to obtain the recombinant plasmid pWPXL-CTDSPL2. The effective siRNA sequence was subcloned into the pLVTHM vector at MluI-ClaI site to get recombinant plasmid pLVTHM-CTDSPL2 RNAi. The recombinant plasmids pWPXL-CTDSPL2 or pLVTHM-CTDSPL2 RNAi were co-transfected with packaging plasmid (pSPAX2) and envelope plasmid (pMD.2G) into 293T cells by calcium phosphate. The efficiency of transfection was confirmed by green fluorescence under fluorescence microscopy 48 h after transfection. The medium was collected by centrifugation (3000 rpm, 5 min, room temperature) 72 h after transfection and then filtered (0.45 μm). The viruses were concentrated by centrifugation (26000 rpm, 2 h, 4°C). The supernatant was discarded and the pellet containing the viruses was re-suspended in serum-free medium. The virus concentration was determined by infecting 293T cells followed by FACS fluorescence analysis.

For gene transduction into CD34+ HPCs, 2 × 10^6 ^viral particles were preloaded onto a RetroNectin-coated plate (Takara, Dalian, China) and incubated at 37°C for 5 h. Just prior to infection, the viral supernatant was discarded and the plate was washed with PBS [28]. Approximately 1 × 10^5 ^CD34+ cells were then added to the preloaded viral plate with growth medium, and the plate was incubated at 37°C. Seventy-two hours after lentiviruses infection, infected CD34+ cells were induced to erythroid differentiation by Epo for a week.

### RNase protective assay

Human globin mRNAs were analyzed by quantitative RNase protection assays. Four plasmids containing the pT7 promoter and a fragment of each of the genes were used as probe templates. pT7ε, pT7γ, pT7α, pT7β-actin were acquired by cloning EcoRI-KpnI fragments of these genes downstream from the pT7 promoter in pcDNA3.1+, linearized with XbaI, giving a protected fragment derived from exons of their mRNAs of 231 nucleotides, 169 nucleotides, 149 nucleotides, 121 nucleotides respectively. pT7β came originally from Dr. Li at the University of Washington [17]. RNA probes, labeled with α-p32 UTP, were obtained by *in vitro *transcription of the plasmids with T7 polymerase and purified according to the manufacturer's instructions (Promega, Madison, WI, USA). Total RNA (4 μg) was hybridized with 3 × 10^5 ^cpm of each probe at 90°C for 2 min and then incubated for 12-16 h at 56°C. RNA samples were then digested with RNase A and T1 for 45 min at 30°C and incubated with Proteinase K for 15 min at 37°C, using the ribonuclease protection assay kit (BD Bioscience, Franklin Lakes, USA). Protected fragments were purified by extraction with chloroform and precipitation with ethanol. Then the samples were denatured at 90°C for 3 min before separation on a 4.75% acrylamide/8 M urea gel at 85 W on the gel sequencing board (Bio-Rad, California, USA). Gels were analyzed with the Cyclone Storage Phosphor System (Packard, USA) and then exposed for 6 to 12 h at -70°C on X-film (Kodak, P.R China).

### Statistical analysis

Statistical analysis was performed using Student's paired *t *test, *p *values less than 0.05 were considered to be significant. All data were expressed as mean ± S.E, and statistical analyses were performed by SPSS version 10.0.

## Authors' contributions

YNM made substantial contributions to design, acquisition of data, analysis of data and drafted the manuscript. XZ participated in the design of the study and carried out DDRT-PCR. HCY performed the Western blot analysis. JWZ conceived of the study, designed and directed the research, helped to analysis data and to draft the manuscript. All authors read and approved the final manuscript.
